# Personalized Nutrition for the Enhancement of Elite Athletic Performance

**DOI:** 10.1111/sms.70044

**Published:** 2025-03-31

**Authors:** Shaun Sutehall, Yannis Pitsiladis

**Affiliations:** ^1^ Clincial Research Division Alder Hey Children's NHS Foundation Trust Liverpool UK; ^2^ Research Institute for Sport and Exercise Sciences Liverpool John Moores University Liverpool UK; ^3^ Department of Sports and Health Sciences Hong Kong Baptist University Hong Kong SAR China; ^4^ International Federation of Sports Medicine Lausanne Switzerland; ^5^ European Federation of Sports Medicine Associations Lausanne Switzerland

## Abstract

Enhancing athletic performance through the manipulation of nutritional intake has ancient roots, with early guidance from “philosophical giants” like Hippocrates, who describes the balance between diet and exercise. Modern sports nutrition emerged in the 20th century, with research identifying carbohydrate (CHO) intake as beneficial for endurance. Studies like Gordon's in the 1920s linked blood glucose levels to marathon performance, while Cade's research in the 1960s on fluid and electrolyte intake led to the founding of Gatorade and the shift toward drinking during exercise to allegedly prevent dehydration and improve sporting performance. Today, sports nutrition is in a “holding pattern” after significant developments in the 1980s, 1990s, and the 2000s. A new era will involve personalized nutrition, but this development will require a game‐changing injection of momentum, recognizing that athletes' responses to nutrition interventions vary widely. New technologies will also need to be developed and perfected, including wearables for real‐time biometric monitoring (e.g., heart rate variability, glucose, and sweat composition and rate), which offer potential for tailored nutrition (i.e., diet and hydration) strategies. Applications of genetic and multi‐omics technologies (like genomics, transcriptomics, metabolomics, proteomics, and epigenomics) are needed to unlock the potential of personalized sports nutrition by analyzing individual responses to factors such as sleep, nutrition, and exercise. The future lies in fast integration of all available data using next‐generation bioinformatics and AI to generate personalized recommendations, with an emphasis on empirical evidence rather than solely commercial interests. As technology matures, sports (and exercise) nutrition will continue refining its practices but will need a paradigm shift to deliver precise interventions that may offer athletes the crucial edge needed to maximize performance while promoting short‐term and long‐term health.

The enhancement of human exercise performance and health through the manipulation of nutritional intake is an age‐old pursuit, with texts describing the range of opinions regarding the optimum diets for exercise more than 2000 years ago (e.g., [1]) and as follows:

Positive health requires a knowledge of man's primary constitution and of the powers of various foods, both those natural to them and those resulting from human skill. But eating alone is not enough for health. There must also be exercise, of which the effects must likewise be known. The combination of these two things makes regimen, when proper attention is given to the season of the year, the changes of the wind, the age of the individual, and the situation of his home. If there is any deficiency in food or exercise, the body will fall sick. Hippocrates c. 460 – c. 370 BC

Despite the prolonged interest in this field, the development of impactful and evidence‐based nutritional practice during sport and exercise remains aspirational rather than reality. The modern discipline of sports nutrition has advanced significantly since the days where athletes would use wine, egg whites and strychnine (a rat poison that could be used as a nerve stimulant) to improve marathon performance [2] are long gone. When examining the historical progression of sports nutrition from ancient times to the present, its cyclical nature becomes evident. As highlighted by Hippocrates, early approaches emphasized individualization (e.g., “… knowledge of man's primary constitution …”), focusing on the unique physiological needs of each athlete. Over time, this perspective shifted toward identifying performance‐enhancing nutritional interventions that could be broadly applied across athletic populations. This shift necessitated the use of interventional studies and group‐based analyses to validate findings and establish standardized dietary recommendations. However, in recent years, there has been a resurgence of interest in personalized nutrition, driven by advancements in wearable technology, biomarker analysis, and genetic profiling, enabling more data‐driven, individualized strategies for optimizing athletic performance.

## 1 A “Recent” Sports Nutrition Revelation: Carbohydrate and Hydration

In the 1920s, studies into carbohydrate (CHO) intake during exercise began in earnest. For example, Gordon et al. [3], noted the association between blood glucose and marathon performance and condition of the runners at the finish. These authors concluded that the “adequate ingestion of CHO before and during any prolonged and vigorous muscular effort might be of considerable benefit in preventing the hypoglycaemia and the accompanying development of symptoms of exhaustion.” Another classic study by Buskirk and Beetham [4] that studied runners competing in the Boston Marathon and Brighton Road Race (Denver, Colorado, USA) and reported 2.5%–7.4% weight loss in the marathon runners, yet performance decrement did not seem to occur and running pace was maintained essentially constant by each runner until the end of the race. These early pioneering studies for some reason were omitted in subsequent deliberations on drinking recommendations.

Despite the potential and the rapidly developing scientific interest in exercise physiology in the early part of the 20th century [5], it was not until the 1960s and 1970s that the first commercialized CHO beverage for sports was developed and studied [6]. In the study that gave rise to the well‐known company “Gatorade,” Cade et al. [6], studied a group of athletes completing a 7 mile run and found that ingestion of a saline‐glucose solution decreased the likelihood of heatstroke in a hot environment. This study, and others (i.e., [7]), established the dogma of avoiding drinking during sport and exercise prior to 1970, to drink as much as possible in order to mitigate dehydration and prevent heat stroke. It is now well accepted that metabolic rate is the main driver of elevated body temperature during exercise and that sweating is regulated independent of skin blood flow and so independent to the cardiovascular response to exercise. For example, Ladell et al. [8], demonstrated that abstention from water had no effect on sweat rate, until water deficits of more than 2.5 L had been incurred. The more recently accepted view for athletes to drink to individual needs (such as “drinking to thirst” [9]) but the concept that dehydration > 2% body mass degrades exercise performance [10] remains popular even though this concept is not supported by ecologically valid studies [4, 11].

There remains significant skepticism about the validity of the research underpinning the claims the manufacturers make about the effectiveness of some of their products, partly due to sports nutrition being poorly regulated, and research and development being sponsored primarily by the sports nutrition industry [12]. A significant number of studies investigating supplements have been funded by the industry (the authors of this manuscript included, e.g., [13]), and while receiving funding from industry to investigate products does not guarantee positive results, readers may perceive the authors to be biased. Many studies suffer from low ecological validity, performing physiological testing in laboratory conditions and often including participants with demographics that do not reflect the target population. For example, it is unclear what the performance implications are for elite footballers of the 33% improvement in endurance running capacity during prolonged intermittent exercise following drinking a CHO‐electrolyte solution versus a control beverage (8.9 ± 1.5 min vs. 6.7 ± 1.0 min, respectively; *p* < 0.05) (mean ± S.E.M, [14]). The field of sport science is also often criticized for inadequate statistical/methodological rigor [15] and publication bias [16].

Despite the many challenges in the evolving field of sports nutrition, significant progress has been made in understanding CHO ingestion, including optimizing its dose [17], form [18], and content [19] in an athlete's diet and sports drinks. It is important to recognize the multiple human factors that influence the formulation of an optimal CHO intake strategy, such as body mass, metabolic preference (fat vs. CHO oxidation), and gastric emptying rates. These factors also apply to other macronutrients like protein and supplements like creatine. Advancements in metabolic testing, wearable technology, and biomarker analysis are improving the ability to assess an individual's physiological responses to nutrition with increasing precision. While these tools offer potential for tailoring nutritional strategies, their full impact on elite sports is yet to be realised. Factors such as data reliability, practical application in real‐world training environments, and the need for continuous monitoring mean that truly individualized approaches remain a work in progress. However, as research and technology evolve, the feasibility of implementing highly personalized nutrition strategies at the highest level of sport is likely to improve over time; this will require a concerted effort by the field of sports nutrition and a modernization seen in other scientific disciplines (e.g., cancer research).

While CHO has been a popular macronutrient to investigate, with multiple consensus statements made (e.g., [20, 21]), there has also been significant interest in identifying, evaluating, and promoting the use of other micronutrients that may impact exercise performance and health. The latest International Olympic Committee consensus statement on nutritional supplementation for elite athletes describes and cautiously supports the use of caffeine, creatine, nitrate, beta‐Alanine, and sodium bicarbonate for their potential to improve sport performance, under specific circumstances [22] and for the replenishment of micro‐ and macronutrients. There have been a multitude of sport nutrition “innovations” with varying scientific support, over the years from CHO beverages with gel‐forming properties [23], hydrogels [24], methods of inducing hyperhydration [25], various supplements with antioxidant properties [26, 27] and slow nutrient release methods [28]. Greater efforts are now needed to generate new sports nutrition innovations while also further enhancing innovations that have already shown promise of efficacy.

## 2 The Average Athlete

There is no such thing as an “average” athlete and it is well‐known in the field of sports science and sports nutrition that “one size does not fit all.” For example, when acclimating members of the Ethiopian Olympic team (i.e., middle‐ and long‐distance runners) for the hot weather expected at the 2008 Beijing Olympics, the same acclimation strategy resulted in sweat rates ranging from 0.8 L/min to 3.6 L/min (unpublished data). This observation of dramatically different sweat rate in a fairly homogenous group of very elite runners in terms of training and performance status, and tribal ancestry, illustrates the inadequacy of the “average” intervention, or the same intervention for all. Similarly, research in sports nutrition has historically been skewed toward male participants. This underrepresentation persists despite the increasing participation of women in sports, the equal representation of female and male athletes in the recent Paris summer Olympic Games [29] and some 45% female athletes in the Paralympic Games [30], and the recognition of unique nutritional and physiological requirements for the female athletes [31]. The effectiveness of a “one‐size‐fits‐all” approach in sports science, medicine, and nutrition has been widely debated, with critics questioning its ecological validity and applicability to diverse athletic populations. Traditional nutrition guidelines are often based on controlled laboratory studies that fail to replicate real‐world competition settings. For example, there has been a debate ongoing for years on the importance of preventing a ~ 2% body mass loss due to dehydration during prolonged endurance events [32]. Here, the prevailing view is that there are performance‐limiting changes in response to significant dehydration [33, 34] but the accenting view is that such studies [33, 34], suffer from fundamental flaws in their design impacting ecological validity. Specifically, participants, most often non‐elite athletes, are subjected to dehydration protocols that do not accurately reflect real‐life competition scenarios of elite athletes and significantly hinders their laboratory performance. Furthermore, when reviewing the physiological responses to exercise during competition, significant rates of dehydration are often observed, often by the fastest runners [4, 7, 11]. Athletes will differ in sweat rate, sodium loss, fuel oxidation, and energy demands, making standardized recommendations insufficient for optimizing performance. Debates such as these have encouraged the discussion about personalized nutrition [35] for some time; however, only recently has wearable technology advanced sufficiently to allow the individual response to a sports nutrition intervention to be properly observed. Promising solutions such as real‐time monitoring of hydration status, sweat composition, glucose levels, and core temperature. Devices such as continuous CGMs and sweat analysis patches provide individualized data, enabling athletes and coaches to tailor hydration and fuelling strategies dynamically. By integrating such data‐driven approaches, sports nutrition can shift toward more personalized, ecologically valid recommendations, enhancing both performance and recovery.

The sports wearables market size was valued at USD 1.75 billion in 2023 and is anticipated to grow at a CAGR (Compound Annual Growth Rate, the rate at which an investment, revenue, or any other metric grows annually over a specified period) of over 15% between 2024 and 2032 [36]. This surge in health and wellness awareness is fueling a growing appetite for fitness monitoring devices and wearables. These are likely to become a routine part of field and real‐life testing in the near future. Not only can developments be made in wearable technology to improve the training of athletes, but also during competition as well. For example, it is now possible to meaningfully measure sweat composition and sweat rate in real‐time [37], and this information could be used to inform in‐race nutritional intake of fluids, enhancing the effectiveness of the hydration program.

**FIGURE 1:**
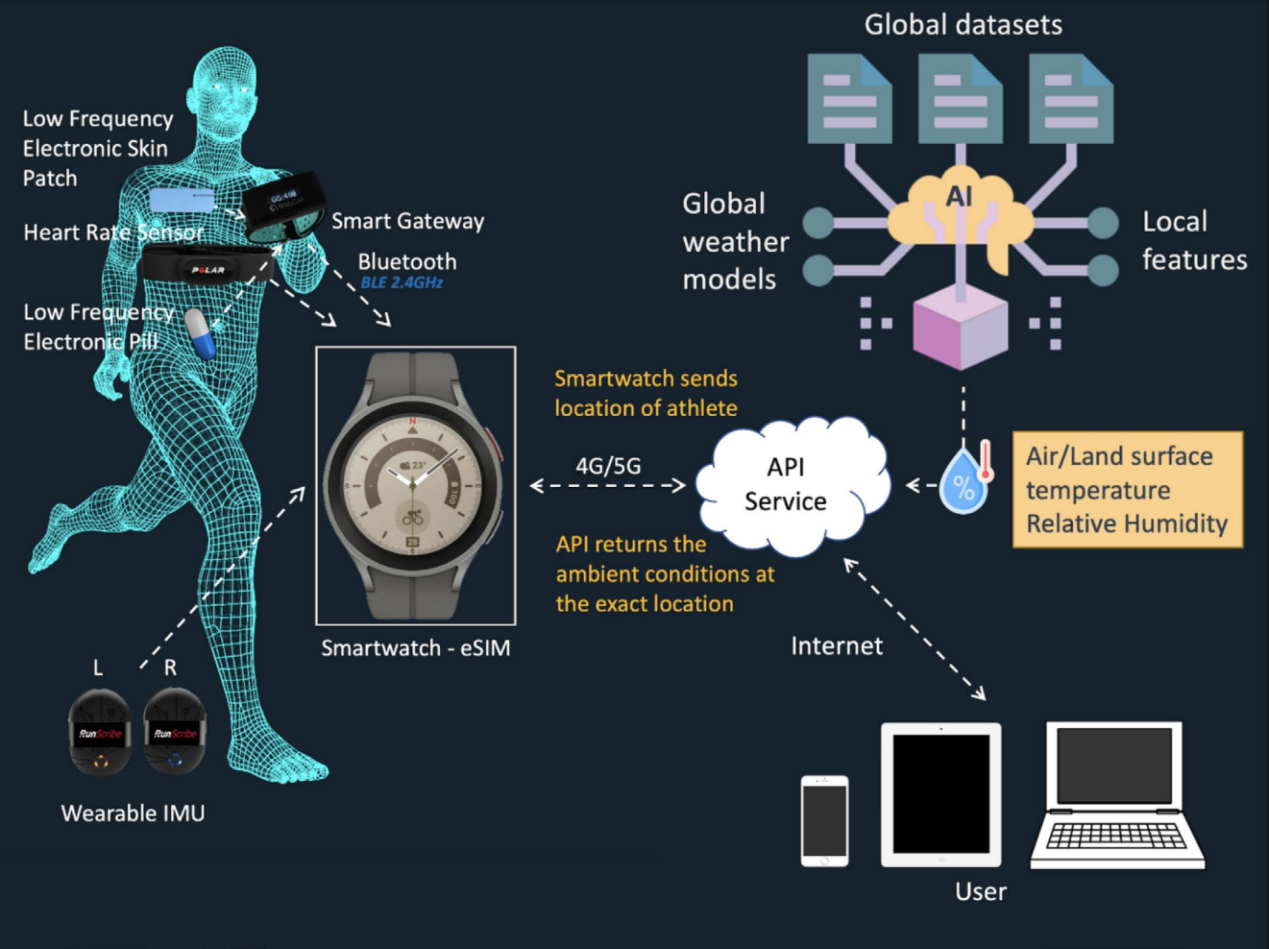
Real‐time biometric and environmental monitoring system used in Paris 2024. AI, Artificial intelligence; API, Application Programming Interface; BLE, Bluetooth Low Energy; IMU, An inertial measurement unit.

Numerous wearable technologies are currently in development and have been piloted in competitions [38, 39] (Figure 1). As we have highlighted [38], an entire “ecosystem” of technology is currently being used to monitor athletes' performance and health metrics. The range of available technologies continues to expand, including ingestible core temperature pills, wearable sweat‐monitoring patches, heart rate monitors, and foot‐worn inertial sensors. Building on this, we previously discussed the rapid advancements in sports technology and the challenges of ensuring these innovations are both effective and ethically applied [39]. We emphasized the importance of striking a balance between leveraging cutting‐edge tools and maintaining fair competition, athlete well‐being, and data privacy. Specifically, we highlight the increasing role of artificial intelligence (AI), real‐time data analytics, and digital biomarkers in refining athlete monitoring, particularly in high‐performance environments [40]. A particularly promising application of these emerging technologies is in the development of personalized nutritional strategies. Continuous glucose monitors (CGMs) allow real‐time tracking of blood glucose fluctuations, providing insights into an athlete's energy availability, metabolic flexibility, and fueling needs during training and competition. This data can inform CHO intake strategies, ensuring optimal glucose levels are maintained to prevent fatigue and enhance performance. Similarly, wearable sweat‐monitoring patches and ingestible electronic pills can provide real‐time data on hydration status, sweat rate, and electrolyte composition. By analyzing an athlete's sweat profile—including sodium, potassium, and fluid loss—practitioners can tailor hydration strategies to prevent dehydration, optimize electrolyte balance, and reduce the risk of heat‐related performance declines. Ingestible thermometric capsules further contribute by continuously monitoring core temperature, helping refine cooling strategies and fluid intake recommendations in extreme conditions. Despite these advancements, we caution that technology alone is not a guaranteed pathway to improved performance [39]. The practical application of these tools depends on accurate data interpretation, integration with individualized training and recovery strategies, and adherence to regulatory and ethical standards. As the field continues to evolve, researchers and practitioners must navigate these complexities to ensure that emerging technologies enhance, rather than disrupt, elite sports. Nonetheless, the ability to collect and apply real‐time physiological data marks a significant step toward a more precise and individualized approach to sports nutrition, hydration, and performance optimization.

A crucial question to consider is what new innovations are needed for personalized nutrition to become a reality and offer more benefits compared to the “one‐size fits all” paradigm? Thankfully, further advancements in technology will help us understand how individuals respond to sports nutrition interventions. For example, to determine the athlete's optimal CHO and fluid recommendations during exercise, we need to consider factors like the energy demands of the activity, cardiovascular strain, metabolite levels, sweat loss, impact of environment, and numerous other factors. Additionally, development of bespoke fit for purpose technology will allow long‐term monitoring of athletes, without the burden of extensive human labour or time costs. To gauge how an individual reacts to different nutritional strategies, repeated testing on the same person in similar and also widely differing environmental conditions will be necessary. The complexity of the sport and the performance environment (i.e., training, minor event, major event) may require more repetitions to accurately assess the impact of an intervention. It is also vital to track athlete adherence to the recommended nutritional plans. This data can help evaluate educational and behavioral change strategies, paving the way for more personalized approaches.

## 3 Genetics‐Based Personalization: A Long‐Awaited Future

Applications in personalized sports nutrition is set to gain significant momentum in the near future, especially with advancements in genomic technologies, like genetic sequencing. It is even suggested that the influence of DNA sequencing could rival that of the microscope [41]. With the rapid advance in the development of omics technologies, such as next‐generation sequencing [42], there is a clear route to develop truly personalized nutrition recommendations. The field of sports nutrition and sport science in general is encouraged to leverage these powerful technologies and stay updated with rapid advancements to enhance the chances of discovering optimal individualized solutions. These technologies are already being used not only in many areas of biomedical research and precision medicine, for conditions like cancer, stroke, and Alzheimer's disease, but also more recently in anti‐doping research [43], offering valuable insights that can be applied to sports nutrition. There exists a growing evidence base describing the transcriptomic, metabolomic, and proteomic responses to nutritional intake/manipulation [44] and practical examples of using metabolomics to better understand the response to exercise in a clinical setting. For example, Hanaoka et al. [45], demonstrated the use of metabolomics to characterize the response to exercise in individuals with cerebral palsy, providing new insights for nutritionists to adapt and improve nutritional guidelines for patients. Our research group has uniquely applied both transcriptomics [46, 47] and metabolomics [48] to the same study and identified potential molecular and metabolomic markers of doping. We are now in the process of combining the analysis with the other omics (including proteomics) to uncover potentially stronger predictive outcomes. This poly‐omics (or “integrative omics”) approach is destined to become common practice in the future, especially if personalized approaches are to achieve greater clinical utility.

Today, however, the use of genetic testing in sports nutrition and science is still in its infancy. The scientific consensus indicates that genetic testing in sports science and sports nutrition currently has limited clinical utility and should not be presently marketed [49, 50]. This stands in stark contrast to the growing number of companies promoting genetic testing with unsubstantiated claims [51, 52]. The global genetic testing market size was valued at $15.5 billion in 2022 and is projected to reach $40.9 billion by 2032, growing at a CAGR of 10.2% from 2023 to 2032 [53]. What is needed is a renewed focus on the traditional, laborious, and costly laboratory experiment, as there is only so much one can learn from meta‐analyses and non‐laboratory‐generated big data. Many research groups worldwide are increasingly relying on non‐laboratory‐controlled data, overlooking the fact that an abundance of poor‐quality data will inevitably generate poor outcomes.

**FIGURE 2:**
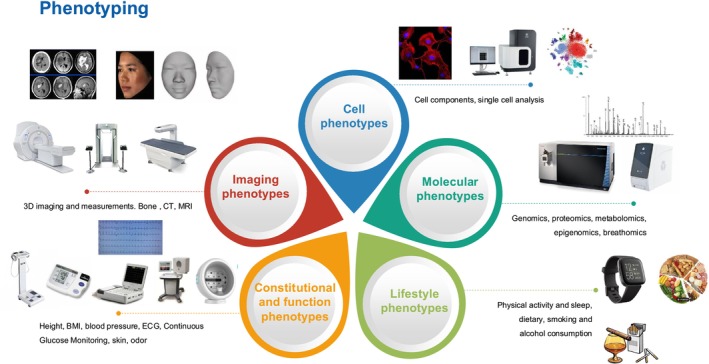
An example of a comprehensive study of the phenotype—“phenomics.”

A significant evolution is also necessary within the emerging field of “phenomics” (the detailed study of the phenotype, e.g., Figure 2), that models health‐ and fitness‐related variables together in a complex system, profiling and modeling an athlete's performance [54], in order to deliver personalized nutrition with greater clinical utility. While genetic testing has long been heralded as a revolutionary tool in sports science and medicine, its full potential remains unrealized due to significant barriers in data acquisition, translation, and practical application [50, 55]. A major challenge lies in the volume and complexity of data generated by OMICS technologies (genomics, transcriptomics, metabolomics, and proteomics) which require advanced bioinformatics for interpretation. Despite rapid advancements in sequencing technologies and biomarker discovery, the integration of these findings into actionable sports nutrition strategies remains slow and inconsistent. For instance, metabolomics can assess how an athlete metabolizes carbohydrates and fats during exercise, enabling personalized fueling strategies. However, the challenge lies in translating this multidimensional data into practical dietary and training recommendations that athletes and coaches can easily implement. Currently, the field lacks a standardized, automated system to integrate and interpret OMICS data in a way that is both scientifically rigorous and user‐friendly. AI and machine learning present a promising solution by enabling the automated processing of vast datasets, identifying meaningful patterns, and generating individualized recommendations. AI‐driven platforms, digital twins, and predictive modeling could bridge the gap between raw genetic data and real‐world application, offering personalized nutrition and recovery strategies based on real‐time physiological needs. Despite these promising advancements, practical implementation remains limited by factors such as data accuracy, regulatory concerns, and accessibility. As research progresses, the key to unlocking the true potential of genetic and OMICS‐based sports nutrition will be more ambitious large studies involving multicenter collaborations so well‐phenotyped samples can be exploited combined with the development of AI‐driven bioinformatics platforms that simplify data interpretation and make precision nutrition a feasible reality for athletes at all levels.

In summary, the limited impact of genomics and OMICS in general in the field of sport science, medicine, and nutrition, as it relates particularly to elite athletes thus far, reflects the early stage of research and methodological challenges rather than a lack of potential. The complexity of human performance traits, small sample sizes, and a lack of understanding of gene–environment interactions have hindered progress. However, genetic testing shows promise in areas like injury risk assessment and personalized training. As research advances, polygenic risk scores and AI‐driven genomic analysis may offer more accurate insights. While genetic findings in the general population may not directly translate to elite athletes, sports‐specific genomic studies hold significant potential.

## 4 Artificial Intelligence and Nutrition

Development of such personalized nutrition will require the application of advanced bioinformatics, including machine learning and AI, to integrate the various layers of biological data for a better understanding of functional outcomes, alongside real‐time assessment of the “phenome” using 5G and 6G technologies, sensors, devices, and applications [40]. 6G (Sixth‐Generation Wireless Technology) is the future of mobile networks, expected to succeed 5G around 2030, promises to significantly impact personalized sports nutrition, sports science, and sports medicine, particularly through its ultrahigh‐speed connectivity, near‐zero latency, and AI integration. With speeds up to 1 Tbps and ultralow latency, 6G will enable real‐time data transmission from wearable devices such as continuous CGMs, sweat analysis patches, and other omics‐based sensors. These devices will offer immediate insights into an athlete's physiological state, such as hydration, glucose levels, and metabolic responses, allowing for instant adjustments in nutrition and hydration strategies. Currently, 3G and 4G networks struggle to provide the necessary bandwidth and low latency for seamless, real‐time data exchange between devices, often leading to delays in data transmission and limiting the ability to act on information instantly. In contrast, 6G will enable AI‐driven networks that enhance data processing and interpretation, allowing for the seamless integration of omics data into personalized nutrition prescriptions. Through AI and machine learning, 6G will support the creation of tailored nutrition plans based on individual biomarker data, improving performance and recovery. Moreover, 6G's global connectivity, even in remote areas, and its potential for autonomous systems will ensure that athletes worldwide have access to cutting‐edge sports science tools and personalized recommendations, advancing both performance and health outcomes in sports. With the ever‐decreasing cost of omics technology and increasing speed at which results can be reviewed, the ambition to see omics technologies routinely applied to sports medicine and sports nutrition settings may soon be realized. Notably, researchers at Stanford University, also working in sports genetics, set the first Guinness World Record for the fastest DNA sequencing diagnostic application, which was to sequence a human genome and provide a medical diagnosis in just over 7 h [56]. Such rapid “sample to result” solutions are now becoming more widely available.

A limiting step in this grand vision may be the speed of data analysis and interpretation of complex omics data. Here, the integration of AI may aid the athlete's support team to identify trends in data and provide tailored advice to the athlete. The use of AI to influence the decision‐making of athletes and support staff is beginning to make significant strides. For example, AI is making significant advancements in sports injury monitoring pathways and enhancing clinicians' ability to monitor and treat injuries effectively [57]. Further research is needed to better understand underlying mechanisms, individual variation, and AI models in order to create personalized nutrition recommendations based also on multi‐omics data [58]. Identifying relevant noninvasive biomarkers is appealing to athletes and practitioners due to the speed and frequency of data collection compared to traditional blood tests or questionnaires. However, it is essential that these technologies are implemented ethically and within established national and international regulatory frameworks, which require further development.

## 5 Conclusion

Athletes relentlessly pursue excellence, risking their bodies for fractions of a second in performance benefit. While the relative impact of nutritional manipulation may only be minor, it might be the fraction of a second or any worthwhile performance advantage that separates winning from losing. The true impact on the performance and health of athletes of truly personalized nutrition over average advice remains to be determined and will require the paradigm shift in the field of sports nutrition advocated here. In this manuscript, the focus has been almost entirely on CHO and fluids, although the important advances in other prominent areas of sports nutrition, such as protein manipulation are equally relevant and could have also been used instead to make the same arguments for the necessary steps needed to achieve personalized nutrition with clinical utility. Furthermore, in our manuscript, we often chose individual endurance sports such as marathon running as an example to highlight advancements and the need for further advancements in sports nutrition, as it is a well‐studied and established model. This choice strengthens our argument by emphasizing the challenges of individualizing nutrition, even in the most researched areas, and underscores the broader need for change in all sports. While the focus is on marathon running, the principles discussed—such as real‐time data, omics, and AI‐driven strategies—apply to other sports like team sports, sprinting, and strength‐based events, making the discussion relevant across diverse disciplines.

## Conflicts of Interest

Y.P. is the founding member of Human Telemetrics (Ltd). The authors have received funding from the following nutrition‐related industry: Maurten AB, Gothenburg, Sweden; Iovate Health Sciences Research Inc., Ontario, Canada; and MarsterFoods, United Kingdom.

## Data Availability

The data that support the findings of this study are available on request from the corresponding author. The data are not publicly available due to privacy or ethical restrictions.
